# Monocyte Count as a Predictor of Major Adverse Limb Events in Aortoiliac Revascularization

**DOI:** 10.3390/jcm13216412

**Published:** 2024-10-26

**Authors:** António Pereira-Neves, Lara Dias, Mariana Fragão-Marques, José Vidoedo, Hugo Ribeiro, José Paulo Andrade, João Rocha-Neves

**Affiliations:** 1 Unit of Anatomy, Department of Biomedicine, Faculty of Medicine, University of Porto, 4050-513 Porto, Portugal; jandrade@med.up.pt (J.P.A.); joaorochaneves@hotmail.com (J.R.-N.); 2 Department of Angiology and Vascular Surgery, Unidade Local de Saúde de São João, 4200-319 Porto, Portugal; lara.romana.dias@gmail.com; 3 Department of Surgery and Physiology, Faculty of Medicine, University of Porto, 4050-513 Porto, Portugal; 4 Cardiovascular R&D Unit, Faculty of Medicine, University of Porto, 4050-513 Porto, Portugal; marianaif.rm@gmail.com; 5 Department of Angiology and Vascular Surgery, Unidade Local de Saúde entre o Tâmega e o Sousa, 4560-136 Penafiel, Portugal; josevidoedo@gmail.com; 6 Community Palliative Care Support Team Gaia, 4430-043 Vila Nova de Gaia, Portugal; hribeiroff@gmail.com; 7 Faculty of Medicine, University of Coimbra, 3004-535 Coimbra, Portugal; 8 Centre for Innovative Biomedicine and Biotechnology, 3004-504 Coimbra, Portugal; 9 MEDCIDS—Faculty of Medicine, University of Porto, 4050-513 Porto, Portugal; 10 Rise@Health, Rua Dr. Plácido da Costa, s/n, 4200-450 Porto, Portugal

**Keywords:** aortoiliac disease, peripheral artery disease, amputation, inflammation mediators, biomarker, hematologic

## Abstract

**Background/Objectives:** Atherosclerosis is a leading cause of death, especially in the developed world, and is marked by chronic arterial inflammation and lipid accumulation. As key players in its progression, monocytes contribute to plaque formation, inflammation, and tissue repair. Understanding monocyte involvement is crucial for developing better therapeutic approaches. The objective of this study is to assess the prognostic value of monocytes for limb-related outcomes following revascularization for complex aortoiliac lesions, thereby emphasizing the central role of monocytes in atherosclerosis. **Methods:** This prospective cohort study-enrolled patients who had undergone elective aortoiliac revascularization at two hospitals between January 2013 and December 2023. Patients with TASC II type D lesions were included, excluding those with aneurysmal disease. Demographic, clinical, and procedural data were gathered, and patients were monitored for limb-related outcomes. Preoperative complete blood counts were analyzed, and statistical analyses, including multivariable Cox regression, were conducted to identify predictors of major adverse limb events (MALE). **Results:** The study included 135 patients with a mean age of 62.4 ± 9.20 years and predominantly male (93%). Patients were followed for a median of 61 IQR [55.4–66.6] months. Smoking history (91%) was the most prevalent cardiovascular risk factor. Preoperative monocyte count >0.720 × 10^9^/L was associated with worse 30-day limb-related outcomes (MALE: OR 7.138 95% CI: 1.509–33.764, *p* = 0.013) and long-term outcomes, including secondary patency (*p* = 0.03), major amputation (*p* = 0.04), and MALE (*p* = 0.039). Cox regression analysis confirmed an elevated monocyte count as an independent predictor of MALE (adjusted hazard ratio 2.149, 95% CI: 1.115–4.144, *p* = 0.022). **Conclusions:** This study demonstrated that patients with a higher absolute monocyte count may be more exposed to the risk of MALE in patients with aortoiliac TASC II type D lesions undergoing revascularization, with predictive accuracy in both the short and long term. Additionally, it was an independent predictor of major amputation. This new marker has the potential to serve as a cost-effective and easily available tool for risk stratification, helping identify patients at higher risk of MALE.

## 1. Introduction

Despite advancements in interventional and pharmacological treatments, atherosclerotic disease remains the leading cause of death in the developed world [1]. This highlights the necessity for developing more effective therapeutic strategies, which requires a more comprehensive understanding of the molecular mechanisms and pathophysiology underlying the disease [2]. Atherosclerosis is defined by persistent low-grade, chronic inflammation within the arterial wall, characterized by the progressive buildup of lipids and inflammatory cells, notably including several subsets of monocyte-derived macrophages, T lymphocytes, and mast cells, within developing atherosclerotic plaques [3]. Blood-derived myeloid cells that infiltrate developing atherosclerotic lesions also comprise monocyte-derived dendritic cells, different subsets of B lymphocytes, and neutrophils, particularly in advanced, frequently thrombotic lesions [4,5].

As understanding of the molecular pathways involved in atherosclerosis advances, the critical role of monocytes in this process becomes increasingly evident [6]. Circulating monocytes attach to and migrate into the vascular endothelial wall, a process enabled by pro-inflammatory cytokines and adhesion molecules [7]. In addition to accumulating lipids and forming atherogenic foam cells, monocytes promote the progression of atherosclerotic plaques by further producing inflammatory cytokines, matrix metalloproteinases, and reactive oxidative species [6,8]. Monocyte subsets are also involved in intraplaque angiogenesis and tissue repair mechanisms [6,8].

The earliest detectable atherosclerotic change is pathological intimal thickening [9], which is considered to be an early surrogate marker for atherosclerosis, while pathological neovascularization is connected to both the early and late stages of the disease [10,11]. Plaque enlargement leads to intraplaque hypoxia, which induces the infiltration of inflammatory cells, subsequently driving local neovascularization [10]. For instance, experimental studies on hypercholesterolemia have demonstrated that adventitial neovascularization in the coronary arteries occurs before the development of plaque protrusion into the lumen [10].

Monocytes, which have been described as key players in the innate immune system, significantly influence the initiation, propagation, and progression of atherosclerosis, as well as plaque thrombosis [2,12]. Experimental data suggest that monocytes are also involved in acute coronary syndromes and post-infarction outcomes; however, human research remains limited [2].

Monocytes’ prognostic value has been previously demonstrated across various medical fields, either as an absolute count or as part of a ratio, and even by subtypes [13,14,15]. For instance, in cancer, the lymphocyte-to-monocyte ratio (LMR), when compared with other inflammatory response markers, had the strongest ability to predict the survival of patients with gastric cancer, with lower values of LMR connected to reduced survival following gastrectomy with a curative intention [13]. Several studies have already been conducted in cardiology, which is more closely related to vascular surgery [16,17]. Monocyte to HDL cholesterol ratio (MHR), an inflammation-based marker, was associated with the severity of coronary artery disease and future cardiovascular events in patients with acute coronary syndrome (ACS) [16]. A recent meta-analysis showed that higher MHR values were associated with higher in-hospital mortality and major adverse cardiovascular events (MACE) in ST-elevated myocardial infarction (STEMI) patients who had undergone a primary percutaneous intervention [17]. Moreover, dynamic changes in monocyte subsets predict MACE and left ventricular function after STEMI [18], and the absolute counts of monocytes are predictive of MACE in patients with coronary artery disease (CAD) [14].

This study aimed to evaluate monocytes’ predictive ability for limb-related outcomes following revascularization due to complex aortoiliac lesions.

## 2. Methods

### 2.1. Patient Selection

Patients who had undergone elective aortoiliac revascularization were consecutively included in a prospective cohort study maintained between two centers, a tertiary hospital and a community hospital, from January 2013 to December 2023. Selection criteria included aortoiliac TransAtlantic Inter-Society Consensus (TASC) II type D lesions, excluding those with aortoiliac aneurysmal disease [19]. The choice between open surgery or an endovascular procedure was taken in a shared decision-making process that included the patient and the surgeon, bearing in mind both the surgeon and the institution’s experience and preferences.

Demographic and clinical characteristics of the patients, including cardiovascular risk factors, procedural details, and lesion-specific information, were obtained through a comprehensive review of their medical records, which also included data from computed tomography scans [20]. Lesion type was described according to TASC II document recommendations [19]. TASC II classifies aortoiliac TASC D lesions as the most complex, including infrarenal aortic occlusion and diffuse iliac disease affecting at least two from the common to the external iliac arteries, such as stenosis of both common and external iliac arteries on the same axis or occlusion of both external iliac arteries.

All aortoiliac procedures were performed using the same type of material to ensure consistency. Specifically, the aortobifemoral grafts that were used were Dacron grafts (Maquet Cardiovascular, model #102687, Aortobifemoral Y graft, 14 × 7 mm and 16 × 8 mm), and this material was consistently applied across all patients. Additionally, the iliac stents were predominantly Cordis SMART stents (Cordis Endovascular, model #SEV-8-100-120, 6–11 mm diameter, 40–120 mm length). Patients were evaluated within the first 30 days following the procedure, as well as during the extended surveillance period. Clinical records from outpatient visits were reviewed to document relevant outcomes, including patient-specific events such as MACE, stroke, and limb-related events, including reinterventions for primary or secondary patency, acute limb ischemia, major amputations, and occlusion without intervention. This study followed the Strengthening the Reporting of Cohort Studies in Surgery (STROCSS) 2019 Guideline [21] and the Transparent Reporting of a multivariable prediction model for Individual Prognosis Or Diagnosis (TRIPOD) guidelines [22]. The study was conducted in accordance with the Declaration of Helsinki, and the protocol was approved by the Institutional revision board/Ethics Committee of Comissão de Ética para a Saúde do *CHUSJ* (Project identification code 246-18 on July of 2018). Each patient’s informed consent was handled accordingly.

### 2.2. Definitions

Data collection followed the Reporting Standards of the Society for Vascular Surgery for lower extremity ischemia [23]. The Rutherford Classification system was employed to categorize the severity and symptoms of chronic lower extremity ischemia [24].

Major adverse cardiovascular events (MACE) were defined as a composite outcome, including acute myocardial infarction (AMI), acute heart failure (AHF), and all-cause mortality [25]. The technical success of the procedure was defined as maintained target vessel patency 24 h postoperatively. Major adverse limb events (MALE) were defined as a composite of reinterventions, including those for primary assisted patency, secondary patency, major amputation of the revascularized artery segment, or occlusion without intervention [26].

### 2.3. Analytical Parameters

All patients had at least one preoperative complete blood count sample available in the two weeks prior to the intervention. When several blood samples per patient met the inclusion criteria, the sample closest to the time of surgery was selected for analysis. Blood analyses were conducted using a Sysmex^®^ XE-2100D (Sysmex Corporation, Kobe, Japan) automated hematology analyzer.

### 2.4. Statistical Analysis

The sample size for a survival analysis for the primary endpoint was determined using online Sample Size Calculators for Designing Clinical Research (https://sample-size.net/sample-size-survival-analysis/; access date: 28 April 2024). The calculation aimed for a statistical power (β) of 90% and an α < 0.05. An estimated sample size of 122 patients was obtained, based on a hazard ratio of 1.8 between groups and an anticipated survival rate of 80% at the conclusion of the follow-up, although higher hazards have been described [20,27,28].

For statistical analysis, SPSS (IBM Corp., released 2023. IBM SPSS Statistics for Windows, version 29.0.2.0, Armonk, NY, USA) was utilized. The Student’s *t*-test was applied for continuous variables normally distributed, with results expressed as mean and standard deviation. For non-normally distributed variables, the Mann–Whitney U test was employed, and results were reported as median and interquartile range. Categorical variables were analyzed using the Chi-squared test. Statistical significance was defined as *p* < 0.05.

To facilitate the interpretation and clinical application of monocyte levels, the authors established a threshold, with the optimal cut-off determined through an optimal binning procedure using MALE as the reference outcome. A multivariate logistic regression analysis was conducted to identify independent clinical and demographic factors associated with 30-day events. The Log Rank estimator was employed to assess the impact of time-dependent variables, and the Wilcoxon test was applied whenever deemed necessary for additional comparisons. Multivariate Cox regression analysis was performed to determine independent predictors of long-term MALE. The backward and forward stepwise regression approach was applied, including variables with a *p* value < 0.10.

## 3. Results

The patients’ demographics and comorbidities are described in Table 1. The cohort included 135 patients with a mean age of 62.4 ± 9.20 years old, with a clear male predominance (93%) and a median follow-up of 61 months (interquartile range 25%–75% [55.4–66.6] months). The most prevalent cardiovascular risk factor was smoking history (current or past), present in 123 patients (91%), followed by dyslipidemia (68%), and hypertension (66%). Diabetes was present in 30% of the cohort. More than 70% of the patients were revascularized due to chronic limb-threatening ischemia (CLTI), while a minority were due to lifestyle-limiting claudication (<30%). Concerning the revascularization method, 43.7% were revascularized through endovascular techniques, while 56.3% were revascularized with open surgery. The mean ABI before revascularization was 0.31 ± 0.13.

When analyzing patients’ demographics and comorbidities concerning monocyte count, the group with monocytes ≤0.720 included 63 patients, while the group with monocytes >0.720 included 72 patients. Patients with a monocyte count >0.72 were older (64.6 ± 8.66 vs. 59.9 ± 9.25 years old, *p* = 0.003) and were more likely to be submitted to endovascular revascularization (*p* = 0.023) when compared to patients with a monocyte count ≤0.72. No other demographic variables or comorbidities differed significantly between the groups. Hematological parameters are described in detail in Supplemental Table S1.

Regarding 30-day outcomes, the only statistically significant difference between the groups was MALE, with patients having preoperative monocyte counts >0.720 experiencing the worst limb-related outcomes (OR = 7.138 CI 1.509–33.764, *p* = 0.013). Even though 30-day MACE and mortality were higher in the group with elevated monocyte counts, the difference did not reach significance (Table 2).

**Figure 1:**
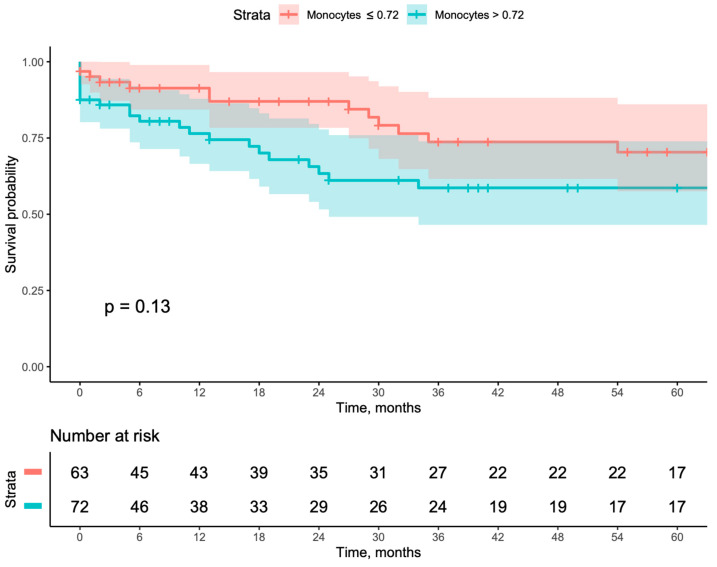
Kaplan–Meyer survival curves and number-at-risk stratified according to a monocyte count of ≤0.72 or >0.72 for primary patency.

**Figure 2:**
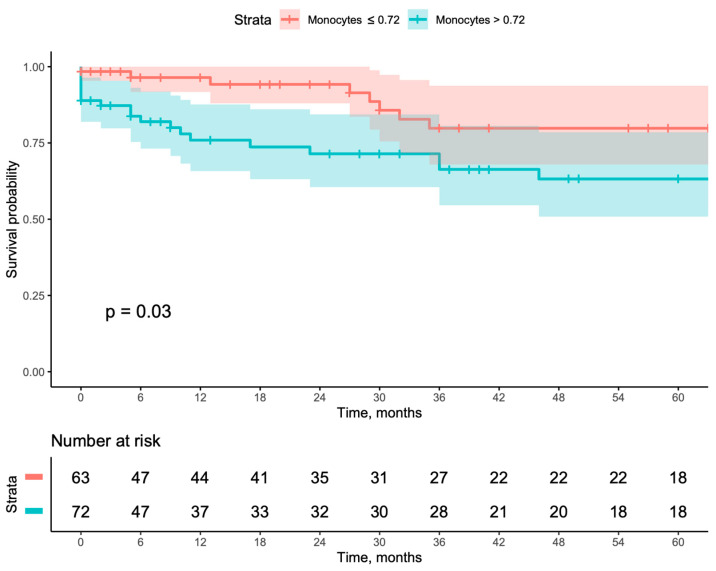
Kaplan–Meyer survival curves and number-at-risk stratified according to monocyte count of ≤0.72 or >0.72 for secondary patency.

**Figure 3:**
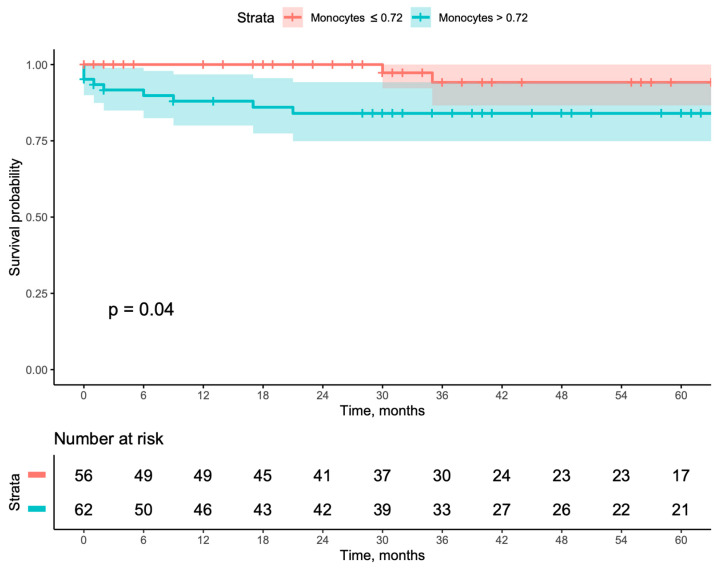
Kaplan–Meyer survival curves and number-at-risk stratified according to a monocyte count of ≤0.72 or >0.72 for amputation.

**Figure 4:**
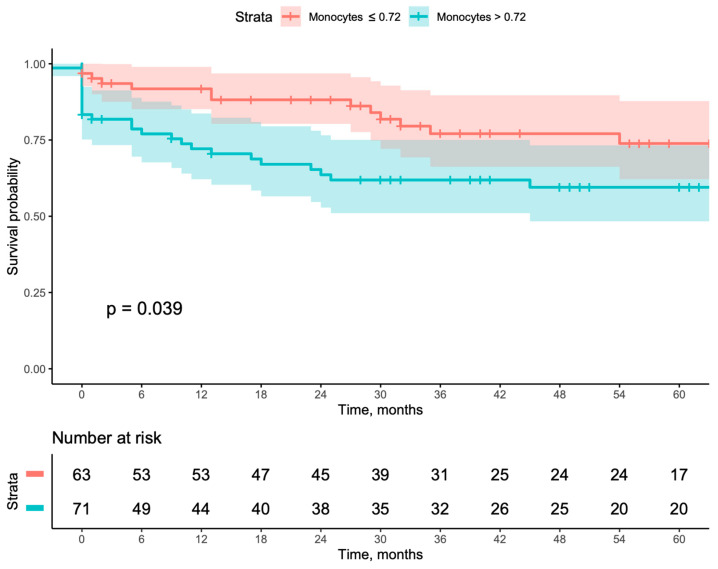
Kaplan–Meyer survival curves and number-at-risk stratified according to a monocyte count of ≤0.72 or >0.72 for major adverse limb events.

When evaluating long-term outcomes using Kaplan–Meier curves, patients with a monocyte count >0.720 had worse outcomes when compared with patients with a monocyte count ≤0.720, including lower secondary patency (*p* = 0.03), higher major amputation (*p* = 0.04), and higher MALE (*p* = 0.039). Even though this same group also showed worse primary patency, it did not reach a statistically significant result (Figure 1, Figure 2, Figure 3 and Figure 4). This result was further corroborated by Cox multivariable regression, with a monocyte count >0.720 being associated with an adjusted hazard ratio (aHR) of 2.149 (95% CI [1.115–4.144], *p* = 0.022) for MALE during follow-up (Table 3).

## 4. Discussion

This study showed that the absolute monocyte count can predict MALE in a cohort of patients with aortoiliac TASC D type II lesions submitted to revascularization. This predictive ability was shown both in the short and long term. Furthermore, it could predict a higher risk of major amputation and lower secondary patency.

Inflammation plays a key factor in the pathogenesis of atherosclerosis [8]. Evidence points out that monocytes seem to have a particular role in atherogenesis. Inflammatory macrophages, the dominant immune cells within atherosclerotic plaques, derive from circulating monocytes that attach to activated endothelial cells and migrate into the intimal layer, as well as from the local proliferation of resident macrophages [29,30]. The importance of monocytes is further highlighted by the observation that lesion generation is significantly reduced when circulating monocytes are inhibited from attaching to endothelial cells [31]. Macrophages drive atherosclerosis progression within the plaque by absorbing oxidized low-density lipoprotein particles (oxLDL) and forming foam cells. Moreover, when stimulated by danger-associated molecular patterns such as oxLDL and proteoglycans present in the atherosclerotic environment, macrophages produce a variety of pro-atherogenic cytokines and chemokines [32]. They also impact plaque stability by regulating collagen production and secreting proteases like matrix metalloproteinases [33,34]. The infiltration of macrophages is a crucial initial step in atherosclerotic plaque formation [35]. The apoptosis or death of these macrophages largely contributes to the formation of a necrotic core in the plaque, with accumulating free cholesterol causing its expansion [36]. Plaque destabilization is a complex process involving an imbalance in the production of inflammatory cytokines, angiogenic factors, and growth factors that promote pathological plaque neovascularization [37]. Matrix metalloproteinases (MMPs) degrade various extracellular matrix proteins, ultimately leading to the rupture of the fibrous cap of the plaque and the formation of an intracoronary thrombus [36]. While the mechanisms driving plaque destabilization are primarily internal to the plaque, circulating peripheral blood monocytes can produce and release mediators that influence key factors involved in this process, such as inflammation, matrix degradation, and thrombogenesis [38]. Additionally, specific monocyte subpopulations have vigorous in vitro angiogenic properties and are a key component of endothelial progenitor cells (EPCs) [25,38]. Therefore, monocytes are crucial in atherogenesis by mediator production, macrophage signaling, foam cell formation, and influencing plaque stability and thrombus formation.

Few studies have assessed monocytes’ impact and predictive ability in vascular surgery, and most existing research utilizes monocytes in ratios. In a cross-sectional study of 216 Type 2 Diabetes (DM) patients without PAD and 218 Type 2 DM patients with PAD, the levels of monocyte/HDL ratio (MHR) were higher in Type 2 DM with PAD patients (vs. without PAD), and they were independently linked with its clinical severity [39]. In this study, although it did not reach statistical significance, patients with elevated monocyte count also presented with worse clinical grades as assessed by the Rutherford Classification. Even though monocyte count was not able to reach statistical significance in predicting primary patency (*p* = 0.13), it showed a predictive ability in secondary patency (*p* = 0.03) during follow-up. The relationship between monocyte count and patency has also been highlighted in vascular access by Caifang Li et al. [40]. This study included 120 patients who submitted arteriovenous fistula (AVF) construction and divided them into three groups based on monocyte count. After a median follow-up of 20 months, 31 AVF failure events occurred. Patients with a baseline monocyte count ≥0.51 × 10^9^/L had the lowest patency rate of AVF. After adjusting for primary clinical data and biochemical indicators, there were statistically significant differences in the AVF patency rates of the three groups (hazard ratio [HR]: 2.774, 95% CI [1.092–7.043]).

The role of immune cells, particularly monocytes and T cells, in the context of peripheral arterial disease (PAD) has been the focus of extensive research by Pawel Maga et al. [41,42] In a study of 45 patients with Rutherford 3–4 PAD undergoing percutaneous transluminal angioplasty (PTA), the author observed an acute reduction in CD8+ T cell subsets immediately following the procedure. This included a significant drop in pro-atherosclerotic, immunosenescent effector T cells, likely due to their adhesion to the vascular wall at the site of injury. These findings suggest a key involvement of T cells in the early immune response to vascular trauma [41]. In a complementary study involving 61 patients with Rutherford 3–4 PAD, monocyte subpopulations were monitored before and after PTA. The results showed a significant reduction in monocyte counts post-procedure, with only subtle signs of monocyte activation, primarily indicated by changes in CD45RA and β-integrins. Interestingly, no direct correlation was found between these monocyte changes and long-term clinical outcomes [42]. These studies highlight the acute immune responses to PTA in PAD, while suggesting that these immune markers may not serve as reliable predictors of long-term adverse events. On the other hand in alignment with the results of this study, in a retrospective study with a cohort of 203 patients submitted to revascularization due to PAD, the cut-off value for the prediction of amputation was 2.55 for the lymphocyte-to-monocyte ratio (LMR) (LMR; sensitivity, 56.25%; specificity, 66.88%) with a decrease ratio being associated with a higher rate of amputation after revascularization [43]. Moreover, in patients undergoing embolectomy for acute lower extremity ischemia, patients with a higher MHR have a significantly higher amputation rate, with MHR being an independent predictor on multivariate analysis (HR: 1.547, CI [1.003–2.387], *p* = 0.04) [44].

A higher circulating monocyte count may reflect not only an increased systemic inflammatory response but also persistent monocyte production, suggesting a broader inflammatory burden that contributes to vascular injury and atherogenesis, thus serving as a potential marker for heightened cardiovascular risk. Monocytes, as representatives of the innate immune system, play a significant role in atherosclerosis initiation, propagation, and progression from a stable to an unstable state [2]. One approach would be to continue investigating the potential presence of various subtypes of monocytes and their specific receptors and roles in cardiovascular disease. Hopefully, this would be useful for developing new therapeutic targets to prevent the progression of atherosclerotic disease and its complications [6].

This study has certain limitations that warrant consideration. Selection bias may have been introduced, as patients with more severe comorbidities and worse clinical conditions, which may not have been fully accounted for by the variables analyzed, could have been preferentially selected for best medical therapy, excluded from revascularization, or directed towards palliative care. Although the findings are valuable, the relatively small sample size of this two-center study, driven by the severity of the selected lesions, limits its generalizability and presents challenges for robust statistical analysis. Furthermore, the predominance of male patients in the cohort restricts the extrapolation of results to the female population, despite their smaller representation in this clinical context. External validation of our findings, particularly the specific monocyte count cut-off, is necessary to ensure broader applicability and confirm the robustness of our results. Furthermore, the detailed survival tables included in the manuscript capture all relevant events, which will aid future meta-analyses of long-term outcomes and provide further insights into the prognostic value of monocyte counts. Another limitation of this study is the lack of longitudinal analysis of monocyte counts, as we only assessed preoperative values. Future research should aim to incorporate both pre- and postoperative monocyte measurements to better understand the temporal dynamics and predictive value of these markers in surgical outcomes.

Conversely, this study’s strengths include its prospective design, extended follow-up period, and homogeneity of the patients’ aortoiliac lesions. By including patients from a large academic teaching institution and a community referral hospital, the external validity of our results is enhanced. However, additional studies are needed to confirm these findings.

## 5. Conclusions

In conclusion, this study demonstrated that patients with a higher absolute monocyte count may be more exposed to the risk of MALE if they have aortoiliac TASC D type II lesions and are undergoing revascularization, with predictive accuracy in both the short and long term. Additionally, it was an independent predictor of major amputation. This new marker can be used as a cost-effective and easily available tool for risk stratification, helping identify patients at higher risk for MALE. Further research is needed to validate this marker further and understand the mechanism of the monocyte effect and its potential for pharmacological manipulation, enabling the individualization of targeted therapy to mitigate the onset, progression, and complications of atherosclerosis in peripheral artery disease.

## Figures and Tables

**Table 1:** Patient’s demographics and comorbidities.

Characteristics	Total *n* = 135 (%)	Monocytes ≤ 0.720 *n* = 63	Monocytes > 0.720 *n* = 72	*p* Value
Age, years (mean ± SD)	62.4 ± 9.20	59.9 ± 9.25	64.6 ± 8.66	0.003
Gender, male	126 (93.3)	58 (92.1)	68 (94.4)	0.580
Hypertension	89 (65.9)	43 (68.3)	46 (63.9)	0.593
Smoking history	123 (91.1)	57 (90.5)	66 (91.7)	0.808
Diabetes	40 (29.9)	22 (34.9)	18 (25.4)	0.227
Dyslipidemia	91 (67.9)	43 (69.4)	48 (66.7)	0.740
CKD	17 (12.6)	7 (11.1)	10 (13.9)	0.627
CAD	37 (27.4)	17 (27.0)	20 (27.8)	0.918
COPD	15 (11.1)	5 (7.9)	10 (13.9)	0.272
CHF	16 (11.9)	8 (12.7)	8 (11.1)	0.776
ASA				0.392
II	53 (39.3)	27 (42.9)	26 (36.1)	
III	74 (54.8)	31 (49.2)	43 (59.7)	
IV	8 (5.9)	5 (7.9)	3 (4.2)	
Rutherford Classification				0.163
III	37 (27.6)	23 (36.5)	14 (18.3)	
IV	53 (39.6)	23 (36.5)	30 (42.3)	
V	36 (26.9)	14 (22.2)	22 (31.0)	
VI	8 (6.0)	3 (4.8)	5 (7.0)	
Endovascular technique	59 (43.7)	21 (33.3)	38 (52.8)	0.023
Open surgery	76 (56.3)	42 (66.7)	34 (47.2)	
SFA disease	83 (61.5)	36 (57.1)	47 (65.3)	0.333
ABI, (mean ± SD)	0.31 ± 0.13	0.33 ± 0.12	0.30 ± 0.14	0.253

Variables are presented as *n* (%) unless otherwise specified. ABI—Ankle-Brachial; ASA—American Society of Anesthesiologists; CAD—Coronary Artery Disease; CHF—Chronic Heart Failure; CKD—Chronic Kidney Disease (creatinine = 1.5 mg/dL); COPD—Chronic Obstructive Pulmonary Disease; SD—Standard Deviation; SFA—Superficial Femoral Artery Atherosclerotic Disease.

**Table 2:** Patients’ 30-day outcomes according to monocyte count.

	Total *n* = 135 (%)	Monocytes ≤ 0.720 *n* = 63	Monocytes > 0.720 *n* = 72	*p* Value
AKI	10 (8.6)	4 (6.7)	6 (10.7)	0.438
ABI Δ, (mean ± SD)	0.45 ± 0.24	0.45 ± 0.241	0.41 ± 0.234	0.398
Rutherford Δ (mean ± SD)	2.5 ± 1.42	2.5 ± 1.39	2.6 ± 1.58	0.826
Prosthetic infection ^#^	7 (5.3)	4 (6.3)	3 (4.3)	0.608
ICU (days) (median IQR)	2 [0–3]	2 [0–3]	2 [0–3]	0.388 *
Enfermary (days) (median—IQR)	8 [3.25–19]	7 [4–11.25]	10.5 [3–24]	0.095 *
MALE	15 (11.1)	2 (3.2)	13 (18.1)	0.006
MACE	11 (8.1)	3 (4.8)	8 (11.1)	0.179
Death	7 (5.2)	2 (3.2)	5 (6.9)	0.324

Variables are presented as N (%) unless otherwise specified. ABI Δ —Ankle-Brachial Index—postoperative minus preoperative; AKI—Acute Kidney Injury; ICU—stay in intensive care unit; IQR—Interquartile Range; MACE—Major Adverse Cardiovascular Event; Rutherford chronic ischemia Δ—preoperative minus postoperative; * Non-parametric test; ^#^ Prothesis infection at 1 year of follow-up.

**Table 3:** Cox multivariable regression proportional hazard ratio for Major Adverse Limb Events.

	Non-Adjusted Hazard Ratios	95% Confidence Interval	*p*-Value	Adjusted Hazard Ratios	95% Confidence Interval	*p* Value
**Age**	1.009	0.976–1.043	0.606	NC		
**Monocytes (*n*)** **>0.720 × 10 ^9^/L**	2.696 *	1.077–6.746	0.034	2.696	1.077–6.746	0.034
	2.149 #	1.115–4.144	0.022	2.149	1.115–4.144	0.022
**Platelets**	1.0	1.0–1.0	0.134	NC		
**Mean Platelet Value**	1.001	0.998–1.005	0.473	NC		
**Preoperative Rutherford**	1.148	0.806–1.636	0.444	NC		
**Open Surgery (vs. endo)**	1.384	0.747–2.562	0.301	NC		

NC—not confirmed. *—continuous variable; #—categorical variable.

## Data Availability

The original contributions presented in the study are included in the article/Supplementary Material, further inquiries can be directed to the corresponding author/s.

## Supplementary Materials

The following supporting information can be downloaded at: https://www.mdpi.com/article/10.3390/jcm13216412/s1, Table S1: Hematological parameters; Figure S1: Kaplan-Meyer notarial tables and number-at-risk stratified according to monocytes count ≤0.72 or >0.72 for primary patency. Figure S2: Kaplan-Meyer notarial tables and number-at-risk stratified according to monocytes count ≤0.72 or >0.72 for secondary patency. Figure S3: Kaplan-Meyer notarial tables and number-at-risk stratified according to monocytes count ≤0.72 or >0.72 for amputation. Figure S4: Kaplan-Meyer notarial tables and number-at-risk stratified according to monocytes count ≤0.72 or >0.72 for major adverse limb events.
