# Supplementary material for: Monocyte Count as a Predictor of Major Adverse Limb Events in Aortoiliac Revascularization
Source: J Clin Med. 2024 Oct 26;13(21):6412. doi: 10.3390/jcm13216412 (PMC11546730; doi:10.3390/jcm13216412)
Supplement: Supplementary file 1 [file jcm-13-06412-s001.zip › jcm-3208037-supplementary.pdf]

**Supplemental Table S1** - Hematological parameters

| | Cohort (n=135) |
|---|---|
| Haemoglobin (g/dl) | 13.1±1.85 |
| MCHC (g/dl) | 33.7±0.99 |
| RDW-CV (%) | 13.9±1.54 |
| RDW-SD (fL) | 46.7±4.96 |
| WBC | 9.4±3.24 |
| Lymphocyte cnt | 2.3±1.09 |
| Lymphocyte % | 26.6±10.89 |
| Neutrophil cnt | 6.0±2.96 |
| Neutrophil (%) | 12.4±17.66 |
| Monocytes x $10^9$/L | 0.7±0.31 |
| Eosinophils | 0.2±0.22 |
| Basophils | 0.1±0.14 |
| Platelet cnt | 266. 1±94.26 |
| MPV (fL) | 10.7±0.8 |
| PDW (fL) | 12.6±1.75 |
| Neutrophil/ Lymphocyte ratio | 2.99±2.02 |

Variables are presented as mean ± Standard deviation unless otherwise specified.

cnt – absolute count; MCHC – mean corpuscular haemoglobin concentration; MPV – mean platelet volume; PDW – Platelet distribution width; RDW-CV — red blood cell distribution width coefficient of variation; RDW-SD — red blood cell distribution width standard deviation; WBC – white blood count.

**Supplemental Figure S1 –** Kaplan-Meyer notarial tables and number-at-risk stratified according to monocytes count ≤0.72 or >0.72 for primary patency.

| **Primary Patency**<br>Log Rank = 0.115<br>Breslow = 0.035 | | 30d | 12 | 24 | 36 | 48 | 60 |
|---|---|---|---|---|---|---|---|
| Monocytes ≤0.720 | Survival (%)<br>SE (%)<br>N<br>events | 96.8<br>2.2<br>57<br>2 | 91.3<br>3.7<br>43<br>5 | 87<br>4.6<br>35<br>7 | 73.7<br>6.7<br>27<br>12 | 73.7<br>6.7<br>22<br>12 | 73.7<br>6.7<br>17<br>12 |
| Monocytes >0.720 | Survival (%)<br>SE (%)<br>N<br>events | 87.3<br>3.9<br>54<br>9 | 76.1<br>5.5<br>37<br>15 | 65.0<br>6.6<br>28<br>20 | 60.3<br>6.9<br>24<br>22 | 60.3<br>6.9<br>19<br>22 | 60.3<br>6.9<br>16<br>22 |

**Supplemental Figure S2 –** Kaplan-Meyer notarial tables and number-at-risk stratified according to monocytes count ≤0.72 or >0.72 for secondary patency.

| **Secondary Patency**<br>Log rank – 0.030<br>Breslow – 0.009 | | 30d | 12 | 24 | 36 | 48 | 60 |
|---|---|---|---|---|---|---|---|
| Monocytes<br><br>≤0.720 | Survival (%)<br>SE (%)<br>N<br>events | 98.4<br>1.6<br>58<br>1 | 96.4<br>2.5<br>44<br>2 | 94.2<br>3.3<br>35<br>3 | 79.8<br>6.6<br>27<br>8 | 79.8<br>6.6<br>22<br>8 | 79.8<br>6.6<br>18<br>8 |
| Monocytes<br><br>>0.720 | Survival (%)<br>SE (%)<br>N<br>events | 88.9<br>3.7<br>56<br>8 | 75.9<br>5.6<br>37<br>15 | 71.5<br>6.1<br>32<br>17 | 71.5<br>6.1<br>28<br>17 | 63.2<br>7.0<br>20<br>20 | 63.2<br>7.0<br>17<br>20 |

**Supplemental table Figure S3 –** Kaplan-Meyer notarial tables and number-at-risk stratified according to monocytes count ≤0.72 or >0.72 for amputation.

| **Major Amputation**<br>Log Rank = 0.040<br>Breslow = 0.018 | | 30d | 12 | 24 | 36 | 48 | 60 |
|---|---|---|---|---|---|---|---|
| Monocytes ≤0.720 | Survival (%)<br>SE (%)<br>N<br>events | 100<br>0.0<br>54<br>0 | 100<br>0.0<br>47<br>0 | 100<br>0.0<br>41<br>0 | 94.2<br>4.0<br>29<br>2 | 94.2<br>4.0<br>23<br>2 | 94.2<br>4.0<br>17<br>2 |
| Monocytes >0.720 | Survival (%)<br>SE (%)<br>N<br>events | 95.2<br>2.7<br>55<br>3 | 88.0<br>4.3<br>46<br>7 | 84.0<br>4.9<br>42<br>9 | 84.0<br>4.9<br>33<br>9 | 84.0<br>4.9<br>26<br>9 | 84.0<br>4.9<br>21<br>9 |

**Supplemental Figure S4 –** Kaplan-Meyer notarial tables and number-at-risk stratified according to monocytes count ≤0.72 or >0.72 for major adverse limb events.

| **MALE**<br>Log rank – 0.052<br>Breslow – 0.013 | | 30d | 12 | 24 | 36 | 48 | 60 |
|---|---|---|---|---|---|---|---|
| Monocytes ≤0.720 | Survival (%)<br>SE (%)<br>N<br>events | 96.8<br>2.2<br>59<br>2 | 91.8<br>3.5<br>53<br>5 | 88.2<br>4.2<br>45<br>7 | 77.1<br>5.9<br>31<br>12 | 77.1<br>5.9<br>24<br>12 | 73.9<br>6.5<br>17<br>13 |
| Monocytes >0.720 | Survival (%)<br>SE (%)<br>N<br>events | 84.5<br>4.3<br>55<br>11 | 73.1<br>5.5<br>44<br>18 | 66.2<br>5.9<br>38<br>22 | 62.8<br>6.1<br>32<br>24 | 60.3<br>6.3<br>25<br>25 | 60.3<br>6.3<br>18<br>25 |
